# Association between DNA damage repair gene somatic mutations and immune‐related gene expression in ovarian cancer

**DOI:** 10.1002/cam4.2849

**Published:** 2020-01-28

**Authors:** Wenjuan Tian, Boer Shan, Yuzi Zhang, Yulan Ren, Shanhui Liang, Jing Zhao, Zhengyi Zhao, Guoqiang Wang, Xiaochen Zhao, Dongxian Peng, Rui Bi, Shangli Cai, Yuezong Bai, Huaying Wang

**Affiliations:** ^1^ Department of Gynecologic Oncology Fudan University Shanghai Cancer Center Department of Oncology Shanghai Medical College Fudan University Shanghai People’s Republic of China; ^2^ The Medical Department 3D Medicines Inc Shanghai People’s Republic of China; ^3^ Department of Obstetrics and Gynecology Zhujiang Hospital Southern Medical University Guangdong People’s Republic of China; ^4^ Department of Pathology Fudan University Shanghai Cancer Center Department of Oncology Shanghai Medical College Fudan University Shanghai People’s Republic of China

**Keywords:** DNA damage repair, immunotherapy, mutation, ovarian cancer

## Abstract

**Background:**

Defects in DNA damage repair (DDR) system may lead to genomic instability and manifest as increased immunogenicity. DDR deficiency is prevalent in ovarian cancer (OvCa); however, the association of DDR mutation with immune profiles in OvCa remains largely unknown. This knowledge will provide an essential basis to the rational design of biomarker‐guided immune combination therapy of OvCa in the future.

**Methods:**

Whole‐exome sequencing data of 587 OvCa from The Cancer Genome Atlas (TCGA) were used to determine the expression profiles of 47 immune‐related genes and the abundance of tumor‐infiltrating immune cells. A Chinese OvCa cohort (n = 220) tested by next‐generation sequencing (NGS) was used to validate the association between DDR status and tumor mutation burden (TMB).

**Results:**

A total of 19.3% in TCGA cohort and 25.9% in Chinese cohort harbored at least one DDR somatic mutation. DDR deficiency exhibited a distinct immune profile with significant higher expression levels of PTPRCAP, CCL5, IFI16, LAG3, IL15RA, and GBP1 in OvCa in the TCGA cohort. Different DDR pathway deficiency displayed various immune profiles. Increased levels of Th1 cells, TMB, and neoantigen were also observed in DDR‐deficient tumors.

**Conclusions:**

DDR deficiency was associated with specific immune signatures in OvCa. Our findings emphasize the urgent need for biomarker‐guided rational immune combination therapy to maximize the OvCa patients who could benefit from immunotherapy.

## Graphical abstract

DDR deficiency exhibited distinct immune profiles in ovarian cancer. DDR deficiency was associated with higher levels of TMB, neoantigen, genomic instability, and improved prognosis in ovarian cancer.

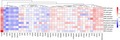

## 1 INTRODUCTION

Ovarian cancer (OvCa) remains the leading cause of death among gynecological cancers.^1^ More than 60% of OvCa patients were diagnosed as advanced stage disease with a 5‐year relative survival of only around 40%.^1^ Despite the standard combined approach based on cytoreductive surgery and platinum‐taxane chemotherapy, most patients ultimately suffered recurrence. Unfortunately, effective options are limited for these patients and therefore novel therapeutic strategies are under urgent need.

Immune checkpoint inhibitors (ICIs) have revolutionized the area of anticancer therapy and been approved for the treatment across a broad range of solid tumors.^2^, ^3^, ^4^ Unfortunately, objective response rate (ORR) is relatively low with ICIs monotherapy in unselected OvCa patients, which was ranging from 9.7% to 15%.^5^, ^6^, ^7^ Current efforts have been focused on the development of new predictive biomarkers to optimize patient benefit from ICIs. PD‐L1 immunohistochemistry diagnostic assay was approved by FDA as a companion to pembrolizumab treatment in non‐small cell lung cancer (NSCLC) patients. However, results from Keynote‐028 have demonstrated that OvCa patients with positive PD‐L1 expression status (levels ≥ 1%) derived limited benefit from pembrolizumab monotherapy (an ORR of 11.5%),^7^ not to mention of challenges such as antibody uniformity, expression heterogeneity, and unstable expression level, which limited the wide use of PD‐L1 in clinical practice.^8^ Although FDA has also approved mismatch repair deficient (dMMR)/microsatellite instability high (MSI‐H) as a biomarker for pembrolizumab treatment in solid tumors, only 3.2% of OvCa harbored dMMR/MSI‐H.^9^ Therefore, promising biomarkers and rational immune combination therapies are needed to maximize patients with OvCa who could benefit from immunotherapy.

OvCa carrying DNA damage repair (DDR) deficiency exhibits a high sensitivity to platinum‐based chemotherapy.^10^ Various DDR pathways dealing with DNA damage were identified, such as mismatch repair (MMR), base excision repair (BER), checkpoint factors (CPF), Fanconi anemia (FA), and homologous recombination repair (HRR).^11^ DDR system is required to maintain the genomic integrity and stability.^12^ Further alteration in DDR may induce a hypermutated phenotype, with a higher tumor mutation burden (TMB), which has been established as a predictive biomarker of ICIs treatment.^13^, ^14^ As an example, defects in MMR (MLH1, MSH2, MSH6, and PMS2) pathway or BER (POLE) pathway, which are responsible for the extraordinary fidelity of human genome, typically exhibited an ultra‐mutated phenotype and further resulted in a durable clinical benefit with ICIs.^15^, ^16^ Melanoma patients with a response to ICIs commonly harbored mutations in BRCA2, a well‐known gene in HRR pathway.^17^ Most recently, a study has revealed that co‐mutations in specific DDR pathways might predict clinical benefit from ICIs treatment.^18^ Another study further verified the association between alterations in 34 DDR genes exhibited a higher TMB level and improved clinical benefit from ICIs in urothelial cancer.^19^ Previous studies have also illustrated the interaction of DDR with immune system, and immune‐related genes such as GZMA may also have an impact on the function of DDR system.^20^ A recent study has revealed that biallelic inactivation of DDR gene mutations exhibits decreased expression of immune regulatory gene in bladder cancer.^21^ Furthermore, DDR inactivation was associated with higher levels of TMB without a higher tumor‐infiltrating immune cell abundance in bladder cancer.^21^

However, a comprehensive view of DDR mutation‐associated immunogenicity in OvCa is still unclear. We hypothesize that DDR mutations may exhibit a distinct immune profile in OvCa. This knowledge will provide essential basis to the design of biomarker‐guided rational immune combination therapy in the future. To validate this hypothesis, we interrogated the expression profiles of 47 immune‐related gene panel using the sequencing data from The Cancer Genome Atlas (TCGA) (n = 587) to characterize the pattern of DDR mutations and investigate their association with the expression profiles of immune‐related genes in OvCa.

## 2 MATERIALS AND METHODS

### 2.1 Characterization of DDR gene status in TCGA cohort

We utilized cBioPortal to retrieve the web‐based whole‐exome sequencing (WES), mRNA expression, and clinical data of 587 OvCa patients from TCGA data portal (://portal.gdc.cancer.gov). A total of 579 patients with DNA mutation data were included into the analysis. Demographic and clinicopathologic characteristics are shown in Table S1. To identify the DDR inactivation mutation status, the DNA data of copy number variant and single‐nucleotide variants of 21 DDR genes (Table S2) were retrieved and combined. We defined alternations in DDR pathway as any non‐synonymous somatic alteration (including missense, nonsense, insertion, deletion, and splice) in protein‐coding region or the presence of homozygous deletions of at least one gene involved in the corresponding DDR pathways.

### 2.2 Chinese cohort tested by next‐generation sequencing (NGS)

A total of 220 ovarian tumors from Fudan University Shanghai Cancer Center (FDCC) and Zhujiang Hospital (ZJH) received next‐generation sequencing (NGS) during January 2016 to July 2019. Genomic DNA from formalin‐fixed paraffin‐embedded (FFPE) tumor specimens or fresh tumor tissues and matched blood sample were used for sequence analysis of 381 cancer‐associated genes (including the 21 DDR genes in Table S2) in the NGS platform illumina Nextseq 500 to > 500X coverage as previously described.^22^ The testing was performed in the College of American Pathologists (CAP)‐certified and Clinical Laboratory Improvement Amendments (CLIA)‐certified 3D Medicines Library.

### 2.3 Analysis of specific mRNA expression profiling

The immune gene signature was defined by 47 immune genes. The immune gene list is shown in Table S3. After data filtering, the mRNA expression data of 40 immune genes were available in the TCGA cohort. The mRNA expression and DNA mutation data were used and the relationships between DDR status and immune gene signature were analyzed. To evaluate the association between immune‐related gene signature and DDR status, we also performed gene set enrichment analysis (GSEA) analysis by javaGSEA 3.0 Desktop Application (://software.broadinstitute.org/gsea/index.jsp). The enrichment score (ES) was the primary result of GSEA and the ES for the gene set was the score at the peak. The positive normalized enrichment score (NES) indicated an association between the DDR status and the gene set enrichment results. A gene set with significant enrichment was identified at false discovery rate (FDR) < 0.05.

### 2.4 Association between DDR gene mutations and genomic changes

To investigate if DDR gene mutations are associated with TMB, DNA somatic mutation data were downloaded from TCGA. TMB was defined as the total number of non‐synonymous somatic mutation in the coding region. We also used the Chinese cohort to validate the association between DDR status and TMB. Germline variants were excluded. The TMB estimated by 381 cancer‐genes NGS panel was strongly associated with TMB estimated by WES.^23^ To determine the association between DDR status and neoantigen, we downloaded the neoantigen data per tumor sample ID from a published study.^24^ The association between DDR status and genomic instability was also explored. Data of genomic instability in terms of number of telomeric allelic imbalances (NtAI), large‐scale state transitions (LST), HRD‐LOH, weighted genome integrity index (wGII), and ploidy status were downloaded from five previously published studies.^25^, ^26^, ^27^, ^28^

### 2.5 Association between DDR mutations and immune cell abundance

To determine the association between DDR status and immune cell abundance, the CIBERSORT algorithm (https://cibersort.stanford.edu) was used to calculate the abundance of 22 immune cell types for TCGA cohort.^29^ The association between DDR status and the immune cells of T helper type 1 (Th1), Th2, and Th17 was also investigated. Data for the three cell types were obtained from a published study.^30^

### 2.6 Associations between DDR mutations and PD‐L1 expression

To determine the association between DDR status and PD‐L1 protein expression level, a total of 175 OvCa with PD‐L1 protein expression data from the Chinese cohort were analyzed. The expression of PD‐L1 protein on tumor cells was evaluated with an immunohistochemical assay (IHC, Ventana, SP263). PD‐L1 expression positive was defined as at least 1% PD‐L1 expression detected on tumor cells or in tumor stroma.

### 2.7 Associations between DDR mutations and clinical prognosis

To determine the association between DDR status and prognosis, clinical data were downloaded from TCGA. Overall survival (OS) was defined as the time from initial surgery to the date of death or last contact (censored). Progression‐free survival (PFS) was defined as the time from initial surgery to the date of progressed or recurrence disease or last contact (censored).

### 2.8 Statistical analysis

Data were analyzed with GraphPad Prism (version 7.01, GraphPad Software, USA), SPSS statistical software (version 20.0, SPSS, IBM Corporation, USA), and R (version 4.3.1, R Development Core Team). Mann–Whitney U test was used to determine the difference between two groups. The Kaplan–Meier method was used to estimate PFS and OS. Differences in PFS or OS were assessed with log‐rank test. Hazard ratios (HR) and associated 95% CIs were determined by Cox's regression. All reported *P* values were two‐sided and considered statistically significant at* P* < .05, unless otherwise specified.

## 3 RESULTS

### 3.1 DDR somatic mutation landscape of OvCa

**Figure 1:**
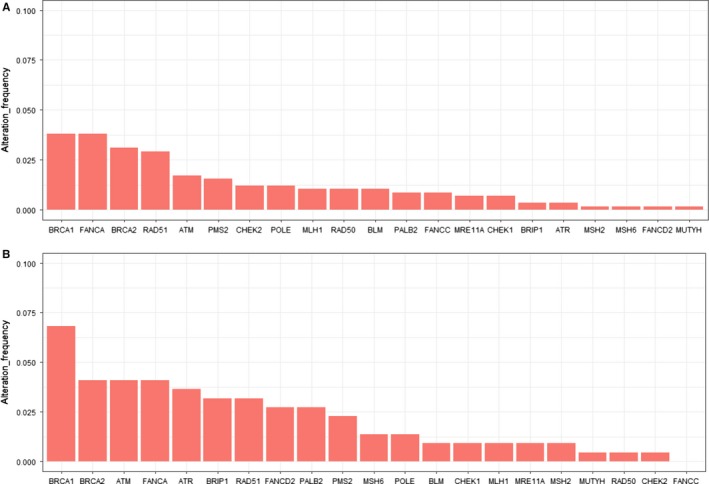
Mutation frequencies of 21 DNA damage repair genes in TCGA cohort and Chinese cohort. (A, B) Alteration frequency of 21 DDR genes in TCGA cohort (A) and Chinese cohort (B)

A total of 19.3% (112/579) OvCa in TCGA cohort tested by WES harbored at least one DDR gene somatic mutation. The frequencies of mutation in FA, HRR, Checkpoint, MMR, and BER pathways were 10.2% (59/579), 8.5% (49/579), 3.9% (23/579), 2.9% (17/579), and 1.2% (7/579), respectively. The frequencies of every DDR gene mutation are summarized in Figure 1. The most frequently mutated genes were BRCA1 (3.8%, 22/579), FANCA (3.8%, 22/579), BRCA2 (3.1%, 18/579), RAD51 (2.9%, 17/579), and ATM (1.7%, 10/579) (Figure 1A).

In the Chinese cohort, a total of 25.9% (57/220) OvCa carried at least one DDR gene somatic mutation. The frequencies of mutations in FA, HRR, Checkpoint, MMR, and BER pathways were 12.3% (27/220), 10.9% % (24/220), 7.3% (16/220), 4.5% (10/220), and 1.4% (3/220), respectively. The most frequently mutated genes were BRCA1 (6.8%, 15/220), BRCA2 (4.1%, 9/220), ATM (4.1%, 9/220), FANCA (4.1%, 9/220), and ATR (3.6%, 8/220) (Figure 1B).

### 3.2 Immune‐related gene expression pattern associates with DDR somatic mutation

**Figure 2:**
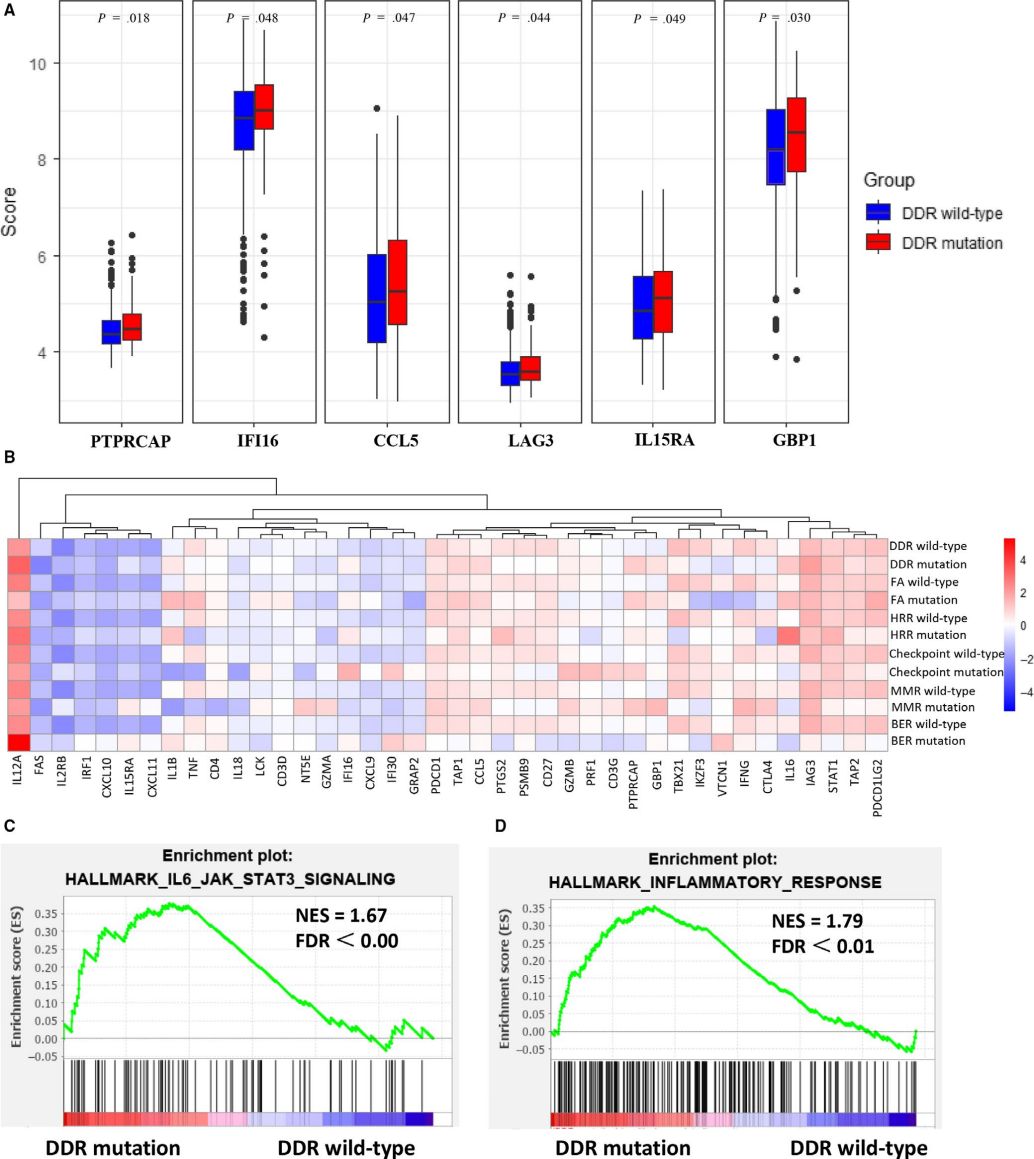
Expression profiles of immune‐related genes in OvCa patients with different DDR deficiency status. (A) DDR deficiency OvCa (red) exhibited significantly higher expression levels of PTPRCAP, CCL5, IFI16, LAG3, IL15RA, and GBP1 compared with DDR wild‐type OvCa (blue). (B) Heatmap depicting the mean difference in immune‐related gene mRNA expression between DDR deficiency and DDR wild‐type in each DDR pathway. (C, D) The GSEA analysis showed prominent enrichment of signatures related to the genes upregulated in IL6‐JAK‐STAT3 signaling (C) and inflammatory response (D) in DDR deficiency OvCa

We further identified 512 OvCa tumors from TCGA, for whom RNAseq and DNAseq data were both available. Among the 40 immune‐related genes, mRNA expression of PTPRCAP, CCL5, IFI16, LAG3, IL15RA, and GBP1 were significantly higher in the DDR mutation group than the DDR wild‐type group (*P* < .05) (Figure 2A). The expression of VEGFA was significantly lower in DDR mutation group compared to DDR wild‐type group. By averaging z‐score expression levels per tumor, tumors with DDR mutation had lower expression levels of CTLA‐4, IFNG, TNF, FAS, and VTCN1, and higher expression levels of IL6, IL1B, and IL12A compared with the DDR wild‐type ones (Figure 2B). Across the five DDR pathway, FA and HRR pathway mutations shared lower expression levels of CTLA‐4 and TBX21, and higher expression levels of IL6, IFI16, and IL12A. Checkpoint, MMR, and BER pathway mutations shared lower expression levels of PDCDILG2, IL6, and CD27. Furthermore, BRCA1 mutation tumors showed lower expression levels of TNF, IL18, and PDCDILG2 compared to BRCA1 wild‐type tumors. However, BRCA2 mutation was associated with a higher expression level of TNF, IL18, and PDCD1LG2 compared with BRCA2 wild‐type tumors (Figure S1).

We further did the GSEA analysis. The results revealed prominent enrichment of signatures related to the genes upregulated in IL6‐JAK‐STAT3 signaling and inflammatory response in DDR mutation group (Figure 2C,D). However, the GSEA‐based analysis did not show a significant prominent enrichment of immunologic signatures in DDR mutation group (Figure S2).

### 3.3 DDR somatic mutations exhibit increased TMB

**Figure 3:**
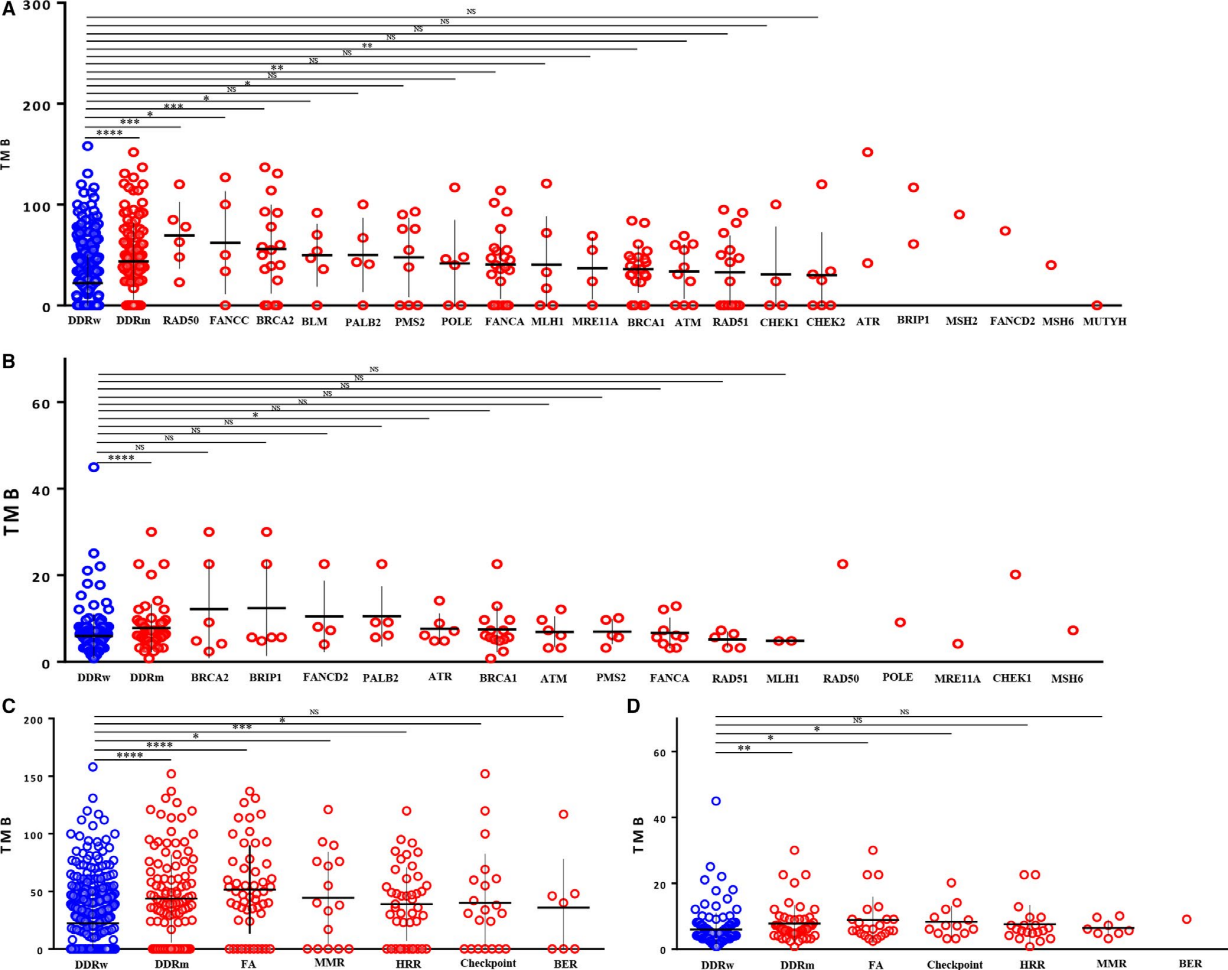
Correlations between DDR gene mutations and tumor mutation burden. (A, B) Comparison of TMB between DDR gene wild‐type (blue) and DDR gene mutation (red) from TCGA (A) and Chinese (B) cohorts. (C, D) Comparison of TMB between each DDR pathway mutation from TCGA (C) and Chinese (D) cohorts. *P < .05, **P < .01, ***P < .001, ****P < .0001, NS, not significant

In concordance with the previous results, a significantly higher level of TMB was observed in OvCa harboring DDR somatic mutations compared to DDR wild‐type OvCa in TCGA cohort (*P* < .0001) (Figure 3). Mutations in one single DDR gene (RAD50, FANCC, BRCA2, PMS2, or BRCA1) also showed a higher TMB level (Figure 3A). More specifically, there was a higher level of TMB in FA, MMR, HR, and Checkpoint pathway mutations than the wild‐type group (Figure 3C).

Similar phenomenon was obtained from the NGS cohort (Figure 3B,D). Although not observed in MMR, BER, or HRR pathway, mutations in FA and Checkpoint pathways displayed a significantly higher TMB level (Figure 3D). Moreover, mutations in ATR gene manifested a significantly increased TMB level in the Chinese cohort (Figure 3B), which was not observed in terms of other DDR gene mutations.

**Figure 4:**
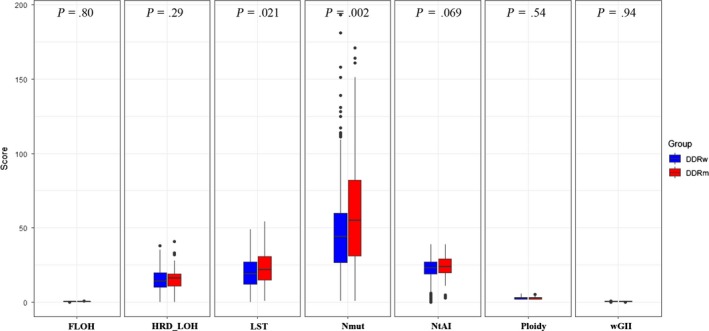
Correlations between DDR deficiency and genomic instability. (a) Comparison of FLOH, HRD_LOH, LST, Nmut, NtAI, Ploidy, and wGII between DDR deficiency (red) and DDR wild‐type (blue) in TCGA cohort. DDR deficiency showed higher levels of LST and Nmut

As expected, tumors with DDR somatic mutations also had higher levels of genomic instability, represented by number of synonymous and non‐synonymous exome mutations (Nmut) (*P* = .002) and LST (*P* = .021), which is in line with the increased TMB levels with DDR mutations (Figure 4).

### 3.4 DDR somatic mutations exhibit increased neoantigen

Given the higher TMB levels in OvCa with DDR mutations, we further investigated whether there was an association between tumor‐specific neoantigen and DDR somatic mutations in OvCa. DDR somatic mutations did show a higher level of neoantigen (Figure S3). Mutations in FANCC, PSM2, PALB2, and RAD51 genes exhibited significantly increased neoantigens. Tumors with FA, Checkpoint, or HRR pathway mutations displayed higher levels of neoantigen compared to tumors with DDR wild‐type.

### 3.5 Association between DDR mutation and immune cell infiltration pattern

**Figure 5:**
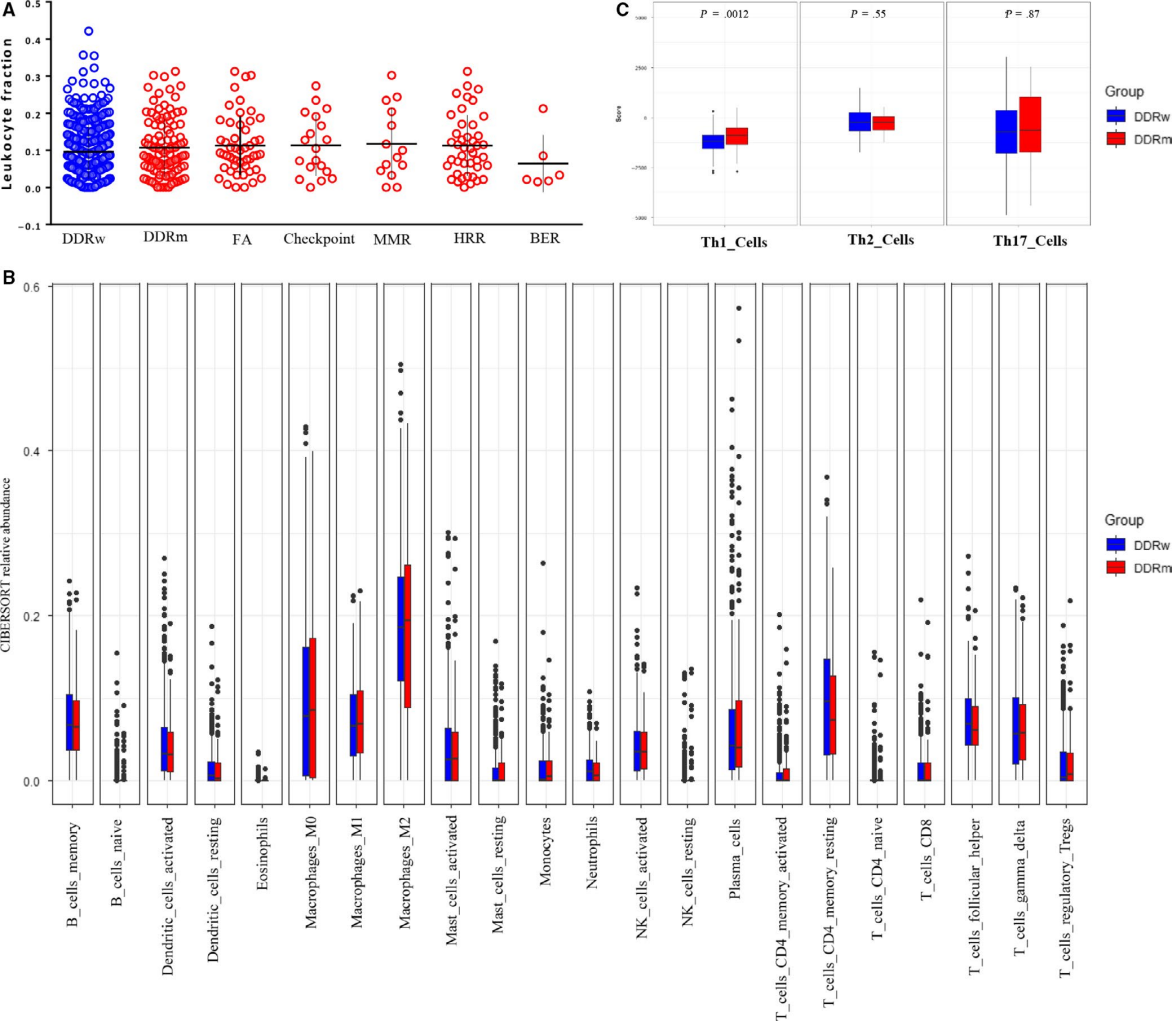
Subtype of immune infiltrates in OvCa. (A) Comparison of leukocyte fraction in each DDR pathway deficiency from TCGA cohort. No significant difference was observed. (B) Comparison of Th1, Th2, and Th17 cells between DDR deficiency (red) and DDR wild‐type (blue) OvCa. OvCa with DDR deficiency (red) showed low abundance of Th1 immune cells compared with DDR wild‐type (blue). (C) Comparison of 22 types of immune cells between DDR mutation (red) and DDR wild‐type (blue) in the CIBERSORT‐based analysis. No significant association was observed

Since higher levels of TMB and neoantigen were observed in DDR mutations, a higher immune cell abundance in DDR somatic mutation was expected. However, based on the CIBERSORT analysis, no association was observed between DDR deficiency and immune cell abundance (Figure 5A,B). Furthermore, DDR somatic mutation exhibited a higher abundance of Th1 immune cells (*P* = .0012) without higher levels of Th2 or Th17 immune cells (Figure 5C).

### 3.6 Association between DDR mutation and PD‐L1 expression

The association between DDR status and PD‐L1 expression was assessed in 175 tumors with PD‐L1 expression data. A PD‐L1 tumor proportion score of 1% or greater was observed for 46.8% (22/47) in the DDR mutation group and 44.5% (57/128) in the DDR wild‐type group without reaching a significant difference level (*P* = .88) (Figure S4).

### 3.7 DDR mutation or higher TMB level shows favorable clinical prognosis

**Figure 6:**
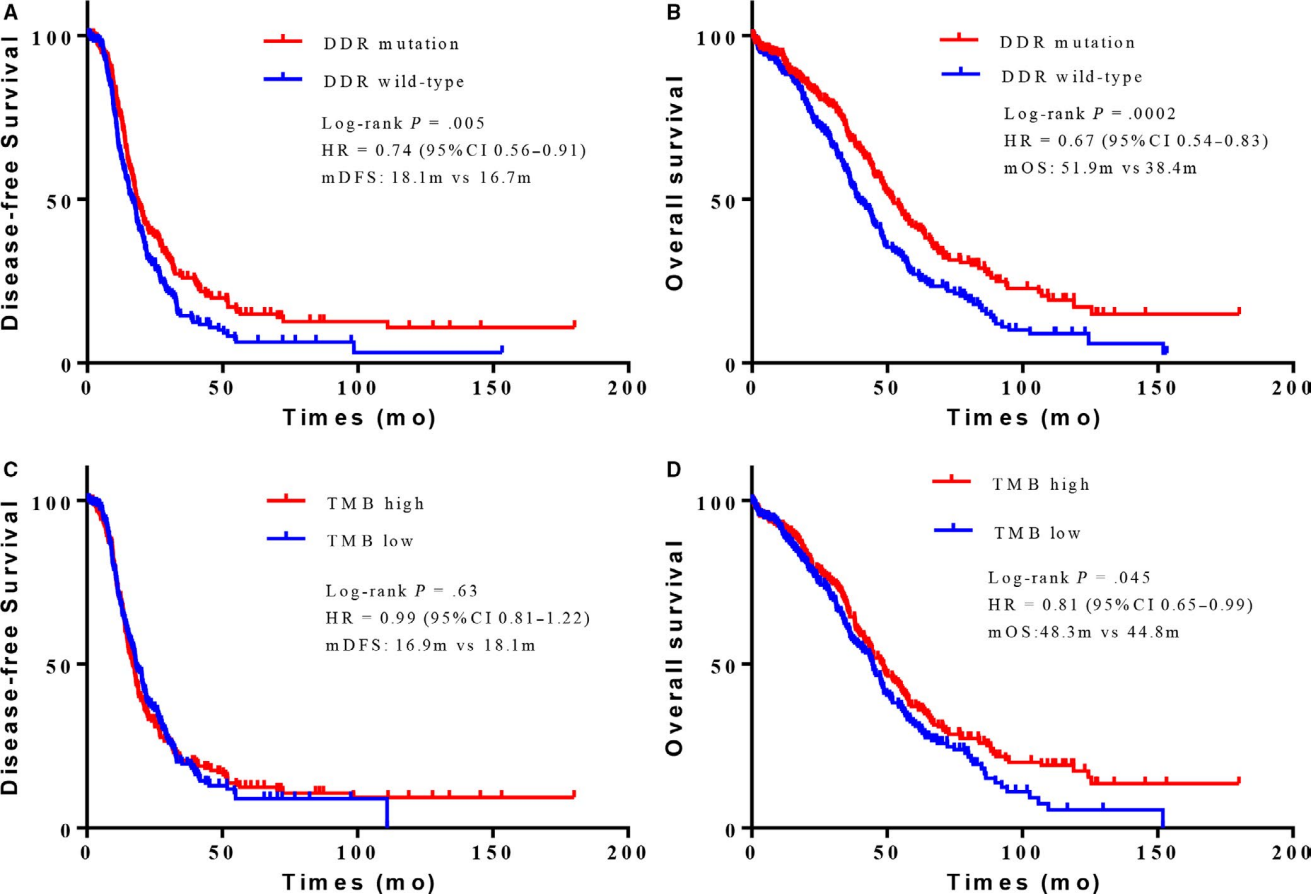
OvCa patients with DDR deficiency or high TMB levels showed favorable clinical outcomes. (A, B) Kaplan–Meier survival curves of PFS (A) and OS (B) comparing DDR mutation (red) with DDR wild‐type (blue) in OvCa patients from TCGA cohort. (C, D) Kaplan–Meier survival curves of PFS (C) and OS (D) comparing high TMB level (red) with low TMB level (blue) groups in OvCa patients from TCGA cohort

Of particular interest, we next investigated whether the 21 DDR genes somatic mutations were associated with improved survival. As expected, the presence of any DDR genes somatic mutation was associated with significantly better OS (median, 51.9 months vs 38.4 months; HR = 0.67, 95%CI 0.54‐0.83; *P* = .0002) and disease‐free survival (DFS, median, 18.1 months vs 16.7 months; HR = 0.74, 95%CI 0.56‐0.91; *P* = .005) in the TCGA cohort (Figure 6A,B). More specifically, patients with mutations in FA pathway had even better OS (median, 51.0 months vs 44.3 months; HR = 0.73, 95%CI 0.58‐0.93; *P* = .019) and DFS (median, 18.1 months vs 17.5 months; HR = 0.75, 95%CI 0.60‐0.95; *P* = .024) (Figure S5I,J). Patients with Checkpoint pathway mutations obtained a significantly prolonged OS (median, 49.6 months vs 44.3 months; HR = 0.76, 95%CI 0.59‐0.98; *P* = .005) (Figure S5C,D). We further assessed the effect of TMB on clinical prognosis. Similar to DDR, patients with a higher TMB level (we used the median as cutoff value) showed prolonged OS (median, 48.3 months vs 44.8 months; HR = 0.81, 95%CI 0.65‐0.99; *P* = .045) (Figure 6).

## 4 DISCUSSION

In this study, we revealed that OvCa with somatic DDR mutation manifested as a distinct immune profile with higher expression levels of PTPRCAP, CCL5, IFI16, LAG3, IL15RA, and GBP1. Increasing levels of Th1 cell abundance, TMB, and neoantigen were also observed in OvCa with somatic DDR mutation.

The presence of DDR mutation has been established as a promising biomarker of immunotherapy in urothelial cancers.^19^ OvCa shows similar characteristics to urothelial cancers, such as an optimistic sensitivity to platinum‐based chemotherapy and a high prevalence of DDR mutations.^10^, ^31^ However, whether DDR mutation could serve as a predictive biomarker of immunotherapy remains unclear in OvCa. In this comprehensive analysis, we explored the association between DDR mutation and immune‐related gene expression in OvCa. Previous studies suggested that DDR deficiency could prime the activation of type I interferons (IFN) system and induce the secretion of chemokines recruiting immune cells, such as CCL5 and CXCL10.^32^, ^33^ Our analyses revealed a higher expression level of CCL5 in DDR mutation OvCa and a higher level of CXCL10 in BER mutation OvCa. DDR mutations were also associated with the activation of IL6‐JAK‐STAT3 pathway. One previous study has shown that DDR deficiency did not exhibit a significantly higher level of immune cells in bladder cancer.^21^ Similarly, we did not identify a significantly higher level of abundance in 22 immune cell types calculated by CIBERSORT. Paradoxically, an increased Th1 cell level was observed in OvCa with DDR mutation, which indicated an immunologically hot state. Th1 cells, which could secrete IL‐2, TNF‐α, and INF‐γ, evolved from CD4 + T cells and stimulated the function of other immune cells, such as macrophage cells and CD8 + T cells.^30^, ^34^ Consistent with the higher levels of Th1 cell abundance, a trend of higher level of macrophage cells was observed in the DDR mutation group. Based on these findings, we speculated that DDR deficiency in OvCa might modulate the response to ICIs to a certain extent, but it might not play a key role. These results also suggested that the expression of proteins such as CCL5, IFI16, LAG3, and others should be evaluated simultaneously, and provided the rationale for future immune combination therapy trials in OvCa with DDR deficiency. Given that PARP inhibitors have shown promising antitumor activity in patients with HRR‐deficient OvCa,^35^ clinical trials aimed at evaluating the efficacy of PARP inhibitor and ICIs combination therapy, such as KEYNOTE‐162, are well expected.

DDR system plays an important role in maintaining genome stability.^12^ TMB, which is considered to be a predictive biomarker of ICIs,^13^, ^36^ may reflect the degree of genomic instability at the nucleotide level. In support of this notion, we discovered a significantly higher level of TMB, Nmut, and LST in DDR‐deficient tumors, which was consistent with previous studies in urothelial carcinoma and melanoma.^17^, ^19^ TMB as a potential predictor may generate different effects on various tumor types. A higher TMB level was closely associated with more neoantigen loads, which have been proven to be the target of ICIs.^8^, ^20^ BRCA1/2 deficiency has been shown to result in a specific signature with higher mutation burden and more tumor‐specific neoantigens in OvCa.^37^, ^38^ In this study, we observed a trend of greater TMB in tumors harboring BRCA2 mutations. Besides, the associations between TMB and DDR gene mutations varied between the two cohorts. The baseline characteristics, such as race and tumor stage, may contribute to the differences. Given that DDR mutations were associated with a higher TMB level and were correlated with improved clinical outcomes in urothelial cancer patients receiving platinum‐based chemotherapy,^19^, ^39^ TMB may be a good prognostic factor in urothelial cancer. In this analysis, we also found that higher TMB was associated with better OS in OvCa, which was consistent with the prognostic role of DDR deficiency in OvCa. It should be noted that, the TMB level of OvCa ranked low in the pan‐cancer analysis.^40^ In other words, although DDR deficiency was correlated with higher TMB, the TMB level in DDR‐deficient OvCa was still far behind the immunologically hot tumors such as lung cancer and melanoma. Further studies are still warranted to investigate whether DDR deficiency is a predictive biomarker for ICIs in OvCa.

Given the methodological differences, it is difficult to compare the overall DDR mutation rate in our study with that in the previous studies. The germline or somatic mutation frequency of 12 DDR gene set provided by cBioPortal (CHEK1, CHEK2, RAD51, BRCA1, BRCA2, MLH1, MSH2, ATM, ATR, MDC1, PARP1, and FANCF) was 39.3% (322/820) in OvCa patients in the public dataset from cBioPortal (://www.cbioportal.org ). TCGA has showed a high HRR deficiency of ~ 50% in high‐grade serous OvCa, including more alteration forms such as BRCA1 hypermethylation, RAD51C hypermethylation, and BRCA1 methylation, which were not assessed in this study.^41^ One previous study has identified the somatic mutations of 13 HRR genes (BRCA1, BRCA2, ATM, BARD1, BRIP1, CHEK1, CHEK2, FAM175A, MRE11A, NBN, PALB2, RAD51C, and RAD51D) in 8.7% (32/367) OvCa patients based on the massively parallel sequencing.^10^ Another study demonstrated a 9.9% (32/324) somatic mutation frequency of 16 HRR genes (ATM, ATR, BARD1, BLM, BRCA1, BRCA2, BRIP1, CHEK2, MRE11A, NBN, PALB2, RAD51C, RAD51D, RBBP8, SLX4, and XRCC2) in OvCa patients from GOG‐0218 clinical trial based on BROCA, a massively parallel sequencing test.^31^ Overall, we showed that 19.3% in TCGA cohort and 25.9% in Chinese cohort harbored at least one somatic mutation in 21 DDR genes in our analysis. Several reasons may explain the difference of mutation frequencies between two cohorts. First, most patients in the TCGA cohort were Caucasian race; however, all patients in our cohort were Asian. Second, most of the tumors in TCGA cohort were stage III and nearly half of the tumors in Chinese cohort were stage IV. Third, previous treatments may exert an effect on the mutation frequency.

One limitation of our study is that we did not distinguish the biallelic or monoallelic inactivation of DDR genes, which might exhibit different degrees of immunogenicity in OvCa. Most of the samples in TCGA cohort were obtained before treatment and the changes of immune‐related gene expression after platinum‐based chemotherapy were ignored. Besides, we defined mutations as non‐silent genomic changes in the coding region including deleterious mutations and other mutations of unknown significance. One prior study has indicated that protein function could still be affected by the benign mutation.^42^ However, we may not be able to identify the specific protein function in this study. Furthermore, germline mutations were not included in this study, which may also have influences on the results.

In conclusion, DDR mutations are prevalent in OvCa and exhibited a distinct immune profile. Our findings may provide insights into the biomarker development for further stratification of OvCa patients and better rational design of immune combination therapies in OvCa in trials.

## CONFLICT OF INTEREST

Drs. Yuzi Zhang, Jing Zhao, Zhengyi Zhao, Guoqaing Wang, Xiaochen Zhao, Shangli Cai, and Yuezong Bai declare that they are employees of 3D Medicines Inc Other authors declare no potential conflict of interests.

## Data Availability

The data that support the findings of this study are available on request from the corresponding author. The data are not publicly available due to privacy or ethical restrictions.
