# Supplementary material for: Association between DNA damage repair gene somatic mutations and immune‐related gene expression in ovarian cancer
Source: Cancer Med. 2020 Jan 28;9(6):2190–200. doi: 10.1002/cam4.2849 (PMC7064027; doi:10.1002/cam4.2849)
Supplement: Supplementary file 1 [file CAM4-9-2190-s001.docx]

**Supplementary Material**

**Table S1. Demographic characteristics**

| **Clinical Features** | **TCGA cohort**  **N=579** | **Chinese cohort**  **N=220** |
| --- | --- | --- |
| Age, median [range], y | 59 [26-89] | 55 [24-82] |
| Race |  |  |
| Asian | 19 (3.4%) | 220 (100%) |
| White | 481 (84.8%) | 0 |
| Black or African American | 34 (5.8%) | 0 |
| Other | 4 (0.7%) | 0 |
| Missing | 41 (5.3%) | 0 |
| Tumor Histology |  |  |
| Serous | 579 (100%) | 211 (95.9%) |
| Clear cell | 0 | 7 (3.2%) |
| Endometrioid | 0 | 2 (0.9%) |
| Tumor Stage |  |  |
| I | 17 (2.9%) | 14 (6.4%) |
| II | 30 (5.1%) | 3 (1.4%) |
| III | 433 (76.0%) | 74 (33.6%) |
| IV | 84 (15.2%) | 100 (45.5%) |
| Missing | 15 (0.9%) | 29 (13.2%) |

**Table S2. Gene list for DNA repair response pathways.**

| **Pathway** | **Genes** [1,2] |
| --- | --- |
| BER (n = 2) | POLE MUTYH |
| Checkpoint (n = 4) | ATM ATR CHEK1 CHEK2 |
| FA (n = 7) | BRCA2 BRIP1 FANCA FANCC FANCD2 PALB2 BLM |
| HRR (n = 4) | BRCA1 MRE11A RAD50 RAD51 |
| MMR (n = 4) | MLH1 MSH2 MSH6 PMS2 |

BER, base excision repair; FA, Fanconi anemia; HRR, homologous recombination repair; MMR, mismatch repair.

[1] Teo MY, Bambury RM, Zabor EC, Jordan E, Al-Ahmadie H, Boyd ME, et al. DNA Damage Response and Repair Gene Alterations Are Associated with Improved Survival in Patients with Platinum-Treated Advanced Urothelial Carcinoma. Clinical cancer research : an official journal of the American Association for Cancer Research. 2017;23:3610-8.

[2] Scarbrough PM, Weber RP, Iversen ES, Brhane Y, Amos CI, Kraft P, et al. A Cross-Cancer Genetic Association Analysis of the DNA Repair and DNA Damage Signaling Pathways for Lung, Ovary, Prostate, Breast, and Colorectal Cancer. Cancer epidemiology, biomarkers & prevention : a publication of the American Association for Cancer Research, cosponsored by the American Society of Preventive Oncology. 2016;25:193-200.

**Table S3. Gene list of immune gene signature.**

|  | **Genes** |
| --- | --- |
| Immune checkpoint | PD-1, PD-L1, PD-L2, LAG3, CTLA4, TIM3, VTCN1 |
| T-effector and INFγ pathway | GBP1, IFI16, IFI30, IFNG, IRF1, STAT1, TAP1, TAP2, FAS, PSMB9, IL15RA, GZMA, GZMB, EOMES, CXCL10, CXCL9, CXCL11, TBX21, PRF1 |
| TCR^*^ | CD27, GRAP2, LCK, PTPRCAP, CCL5, IL2RB, IKZF3, CD3G, CD74, CD3D, CD8A, CD4, TIGIT |
| TME^#^ | IDO1, PTGS2, IL1B, IL18, IL6, IL12A, TNF, CD73 |

*TCR, T cell receptor

#TME, tumor microenvironment

[1] Dong ZY, Zhong WZ, Zhang XC, Su J, Xie Z, Liu SY, et al. Potential Predictive Value of TP53 and KRAS Mutation Status for Response to PD-1 Blockade Immunotherapy in Lung Adenocarcinoma. Clinical cancer research : an official journal of the American Association for Cancer Research. 2017;23:3012-24.

[2] Fehrenbacher L, Spira A, Ballinger M, Kowanetz M, Vansteenkiste J, Mazieres J, et al. Atezolizumab versus docetaxel for patients with previously treated non-small-cell lung cancer (POPLAR): a multicentre, open-label, phase 2 randomised controlled trial. The Lancet. 2016;387:1837-46.

[3] Llosa NJ, Cruise M, Tam A, Wicks EC, Hechenbleikner EM, Taube JM, et al. The vigorous immune microenvironment of microsatellite instable colon cancer is balanced by multiple counter-inhibitory checkpoints. Cancer Discov. 2015;5:43-51.

**
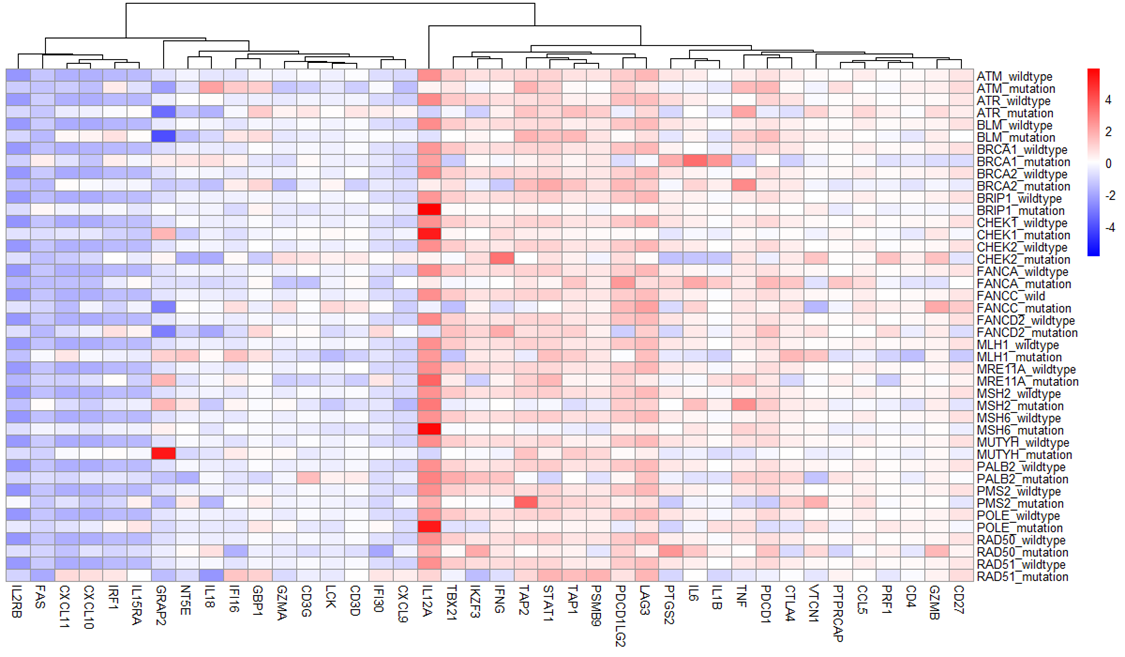
Figure S1. Correlation between DDR genes mutation and immune gene signature.** Heatmap depicting the mean difference of immune gene mRNA signature between DDR gene mutation and DDR gene wild-type.

**
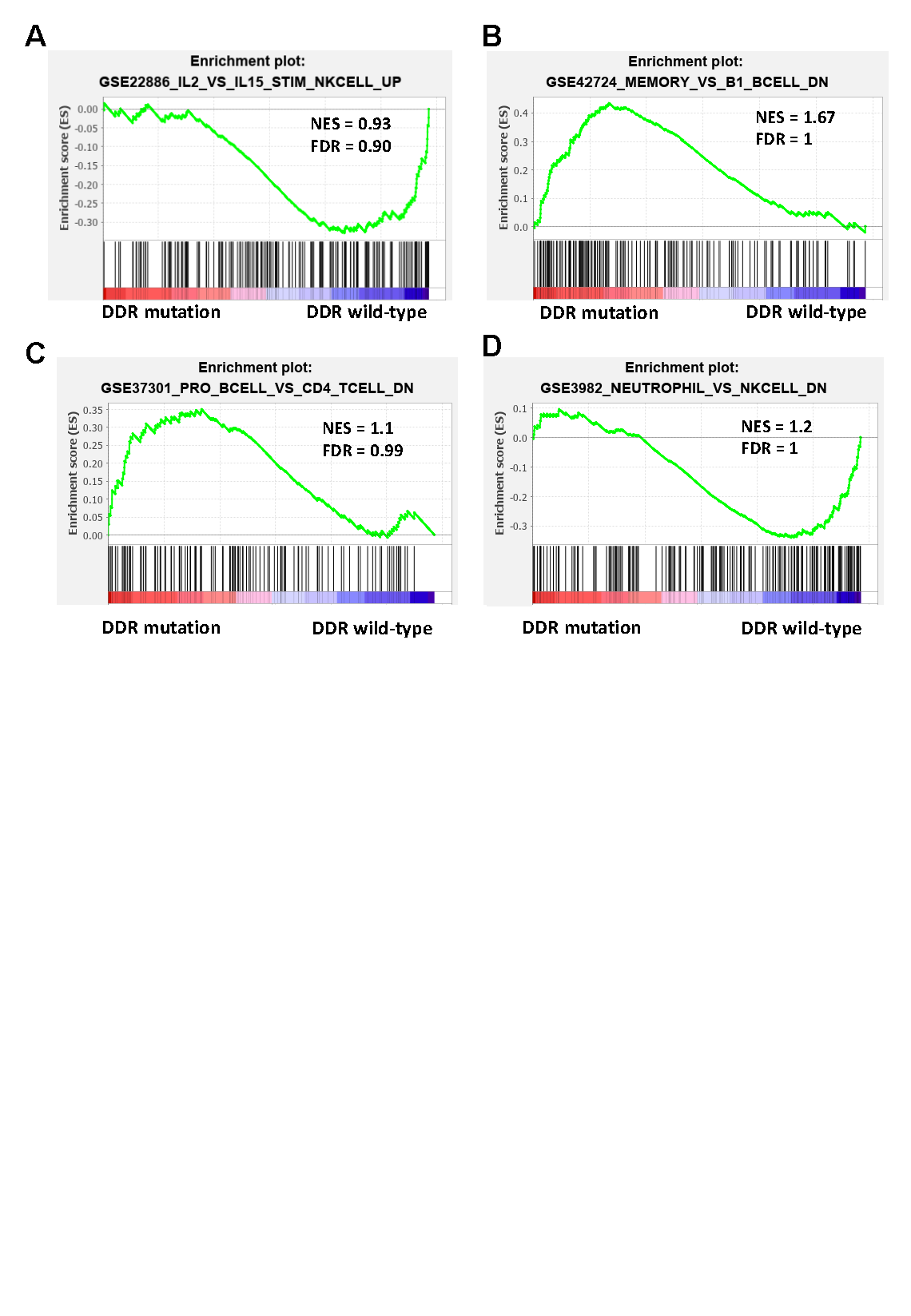
**

**Figure S2.** **Correlation between DDR genes mutation and immune gene signature.** The GSEA-based analysis did not show a significant prominent enrichment of immunologic signatures in DDR mutation group, such as comparison of IL12 versus IL15 stim NK cell up, or memory versus B1 B cell down.


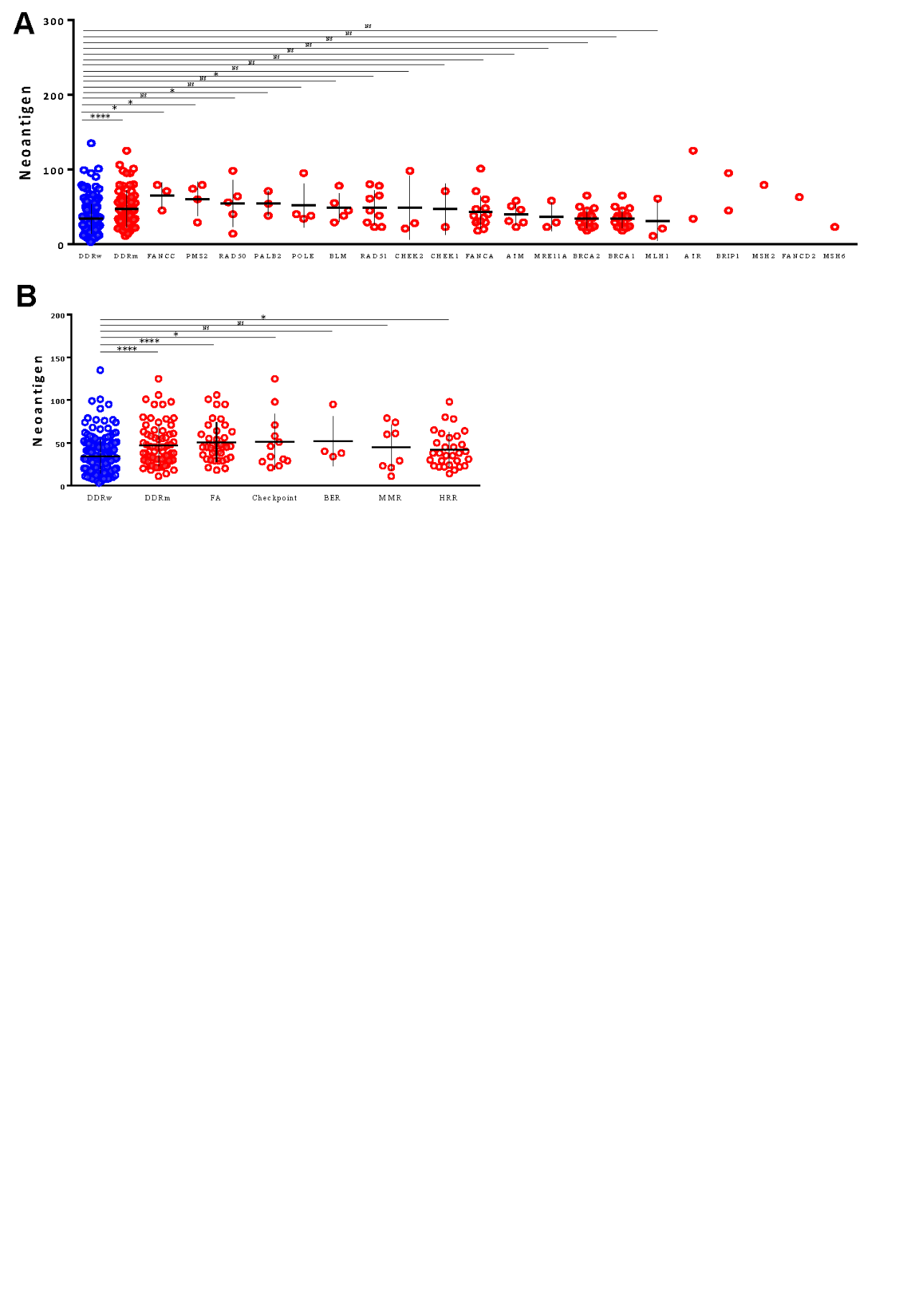


**Figure S3. Correlations between DDR genes mutations and neoantigen.** (A) Comparison of neoantigen between DDR gene wild-type (blue) and DDR gene mutation (red) from TCGA cohort. (B) Comparison of neoantigen between each DDR pathway mutation from TCGA cohort. *P<0.05, **P<0.01, ***P<0.001, ****P<0.0001, NS, not significant.

**
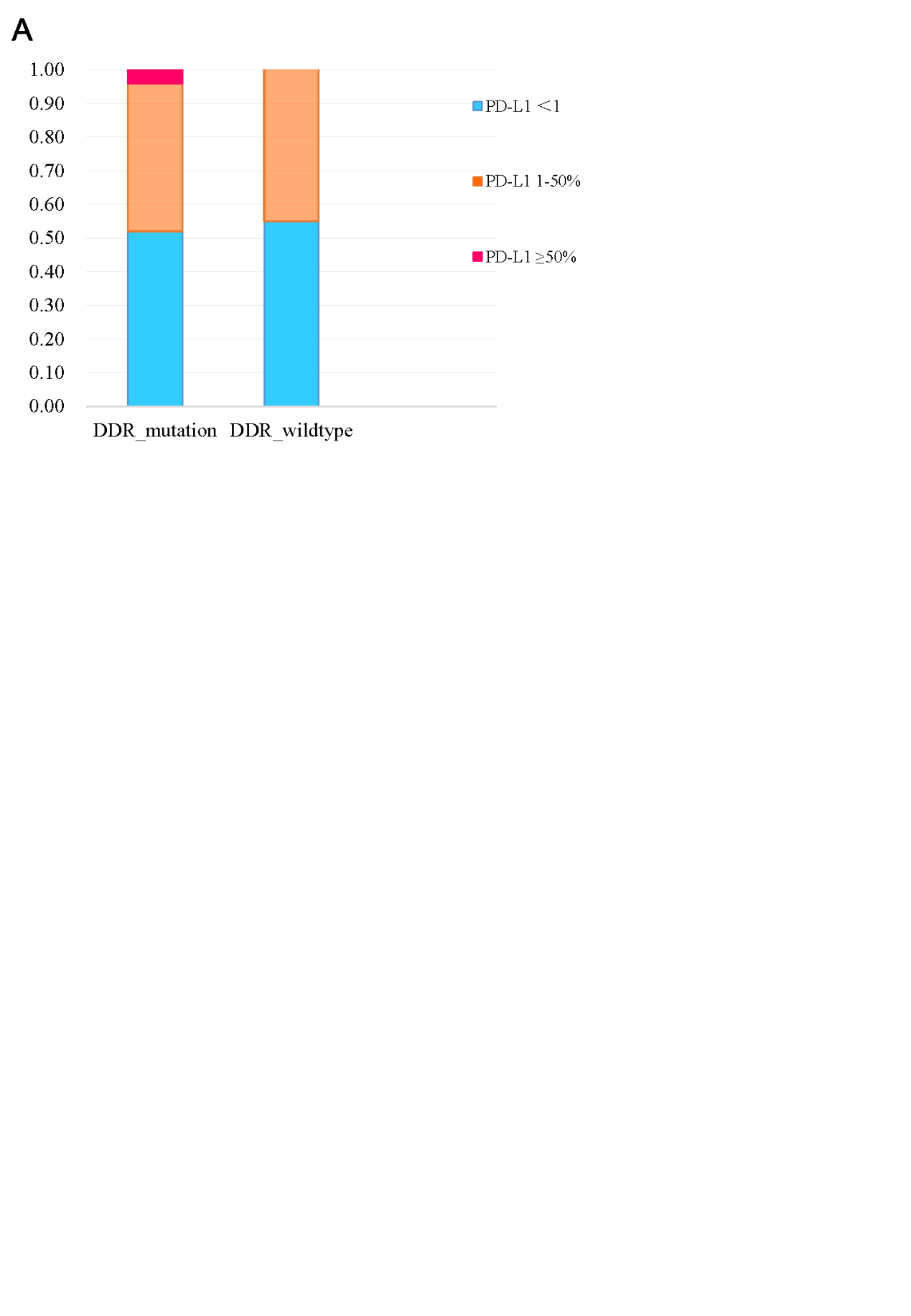
**

**Figure S4. Correlation between DDR mutations and PD-L1 expression level.** Bar chart showing PD-L1 expression level in DDR mutation group and DDR wild-type group.

**
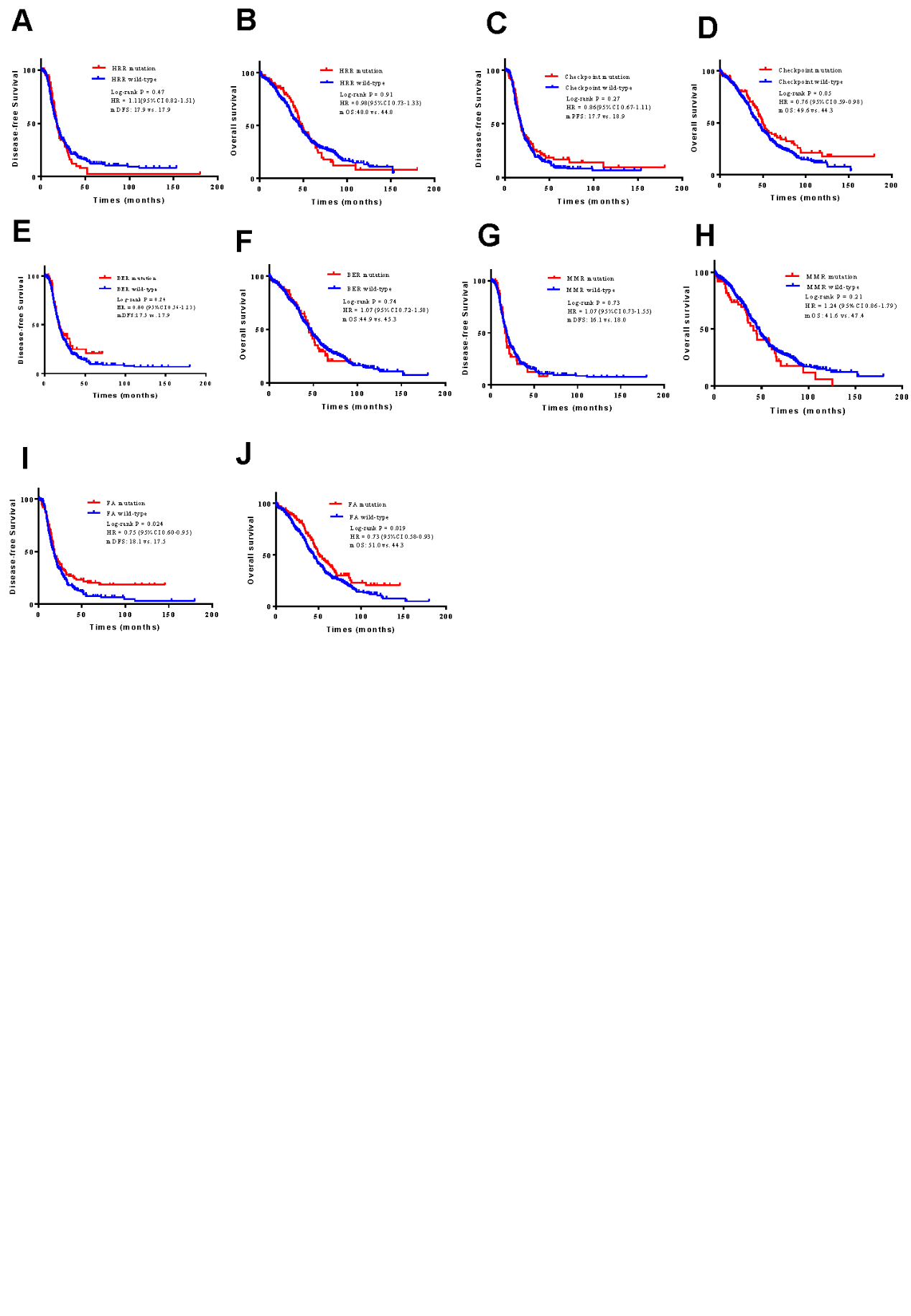
Figure S5. Correlation between DDR mutations in each pathway and clinical prognosis.** (A, C, E, G, I) Kaplan-Meier survival curves of PFS comparing HRR (A), Checkpoint (C), BER (E), MMR (G), FA (I) pathway mutation (red) with DDR wild-type (blue) in OvCa patients from TCGA cohort. (B, D, F, H, J) Kaplan-Meier survival curves of OS comparing HRR (B), Checkpoint (D), BER (F), MMR (H), FA (J) pathway mutation (red) with DDR wild-type (blue) in OvCa patients from TCGA cohort. HRR, homologous recombination repair; BER, base excision repair; MMR, mismatch repair; FA, Fanconi anemia.
